# Overcoming Oncogenic Mediated Tumor Immunity in Prostate Cancer

**DOI:** 10.3390/ijms18071542

**Published:** 2017-07-17

**Authors:** Geoffrey Bryant, Lin Wang, David J. Mulholland

**Affiliations:** Tisch Cancer Institute, Icahn School of Medicine at Mt. Sinai, New York, NY 10029, USA; geoffrey.bryant@mssm.edu (G.B.); lin.wang1@mssm.edu (L.W.)

**Keywords:** immunotherapy, immune checkpoint, prostate cancer, treatment resistance, tumor microenvironment

## Abstract

Immunotherapy is being tested intensively in clinical trials for prostate cancer; it includes immune checkpoint inhibition, prostate specific antigen (PSA) vaccines and dendritic cell-based strategies. Despite increasing evidence for clinical responses, the consensus of multiple trials is that prostate cancers are poorly responsive to immunotherapy. Prostate cancer has a high degree of pathological and genetic heterogeneity compared to other cancer types, which may account for immunotherapeutic resistance. This hypothesis also implies that select types of prostate tumors may be differentially responsive to immune-based strategies and that the clinical stage, pathological grade and underlying genetic landscape may be important criteria in identifying tumors that respond to immune therapies. One strategy is to target oncogenic driver pathways in combination with immunotherapies with the goal of overcoming tumor immunity and broadening the number of patients achieving a clinical response. In this analysis, we address the hypothesis that driver oncogenic signaling pathways regulate cancer progression, tumor immunity and resistance to current immune therapeutics in prostate cancer. We propose that increased responsiveness may be achieved through the combined use of immunotherapies and inhibitors targeting tumor cell autonomous pathways that contribute towards anti-tumor immunity in patients with prostate cancer.

## 1. Introduction

Prostate cancers (PC) are capable of durable clinical responses to immunotherapeutic strategies. This is exemplified by the clinical successes of dendritic cell-based therapies (Sipuleucel T) [1] and prostate specific antigen vaccine-based immunotherapy (Prostvac) [2]. Nevertheless, these treatments typically achieve responses in a minor portion of total patient populations, with <5% of patients achieving a >50% reduction in prostate specific antigen (PSA) [2,3] and a 10–13% increase in the three-year post clinical trial survival rate compared to controls: Prostvac, 30% vs. 17% control [2]; Sipuleucel T, 31% vs. 21% control [4]. Immunotherapeutic strategies targeting immune checkpoints have shown even more limited clinical success in PC patients [5,6], particularly when compared to other cancer types such as melanoma and non-small cell lung cancers [6,7]. Hence, understanding the mechanisms accounting for the lack of clinical response in PC patients is of paramount importance.

One of the unique features of PC is the high degree of tumor heterogeneity whether evaluated at the histological, genetic, or cell signaling level. Heterogeneity presents challenges for cancer treatment, particularly when patients of similar clinical staging can have very different underlying genetic landscapes. Variation in primary tumor composition can pose challenges for standard-of-care treatments, including radiation, chemotherapy and targeting the androgen receptor signaling pathway [8]. Beyond driving cell autonomous disease progression, the accumulation of mutations and oncogenic driver pathways may promote increases in immunosuppressive cells and exhaustion of immune effector cells in the tumor microenvironment (TME) [9,10,11]. Therefore, a greater response to immunotherapy may be achieved through coordinate targeting of key driver pathways relevant to early, mid and late stages of PC. Our hypothesis is best supported by studies in melanoma and triple negative breast cancer, where oncogenic signaling pathways have been shown to be responsible for mediating tumor immunoresistance [9,12,13,14]. Convincing evidence using preclinical mouse models of melanoma demonstrates that pharmacological targeting of oncogenic pathways such as BRAF and PI3K signaling can increase sensitivity to immune checkpoint blockade [14,15]. We suggest that oncogenic signaling drivers contribute towards disease progression and the evasion of immunosurveillance in PC, but may also offer treatment opportunities to lessen immunoresistance and enhance immunotherapies. 

## 2. Prostate Cancer Heterogeneity: A Challenge for Targeted Therapies

Mutationally dominant neoplasms, such as BRAF or RAS driven melanomas [16], are often treated successfully with single agent therapies [17]. However, PC heterogeneity, found even during early and mid-stage disease, suggests that to achieve greater clinical response rates, treatments must be done with consideration of the genetic landscape and oncogenic driver pathways. Longitudinal whole genome and deep sequencing of primary and metastatic tumors have revealed the polyclonal nature of PC [18], and that the expansion of subclones with divergent oncogenic drivers may account for resistance to standard-of-care therapies [19]. Moreover, analysis of metastatic sites in PC patients has revealed the occurrence of inter-metastatic site seeding of subclones harboring distinct driver mutations, accounting for further tumor heterogeneity and treatment challenges [18]. Phylogenetic analysis of longitudinal sequencing studies of PC patient samples from primary and metastatic sites suggests that these changes are driven by increasing mutational divergence of distinct subclonal populations [18,19]. This increase in heterogeneity and polyclonality during the progression of PC necessitates targeted strategies that account for the different cancer drivers in distinct clonal populations within a patient, which may promote both disease progression and tumor immune evasion. Stratifying patients into subtypes through the identification of targetable oncogenic drivers may allow for the development of patient-specific combinations of targeted therapies to inhibit pathways mediated by distinct clonal populations. Such analysis of tumor-specific genomic alterations and gene expression perturbations are likely important for the identification of effective therapeutic combinations in PC patients. These analyses will also require additional strategies to augment current standard-of-care approaches including histological analysis. Developing minimally invasive and cost-effective techniques to classify stage dependent changes in tumors occurring both during progression and as a result of treatment is paramount to the development of more effective treatment combinations. Oncogenic driver-specific disease signatures utilizing biomarkers such as circulating tumor cells (CTCs) [20], extracellular vesicles (EVs) [21], tumor-secreted microRNAs (miRNA) [22], and cell-free DNA (cfDNA) [23] in the blood, urine, and other body fluids through “liquid biopsies” [24,25,26] may provide a non-invasive strategy to stratify patients into treatment-specific subtypes. This is exemplified through the analysis of phosphatase and tensin homologue (PTEN) alterations in PC [27] and P53 changes in triple negative breast cancer [28]. 

### *Progression-Dependent Changes in Oncogenic Driver Pathways* 

During the early stages, PC is predominantly an androgen-dependent disease with most patients being treated with agents that either reduce systemic androgen levels (LHRH agonists) or directly antagonize the androgen receptor (AR) (bicalutamide) [29]. Eventually, cancer cells adapt to castrate androgen levels to form recurrent disease [30]. This progression is frequently mediated through AR reactivation [31], as well as through acquisition of additional alterations such as PTEN loss and PI3K activation [32]. Patients progressing despite androgen deprivation therapy (ADT) may be treated with agents targeting AR signaling, such as the AR antagonist enzalutamide or the androgen synthesis inhibitor, abiraterone acetate. These treatments can delay progression despite the failure of ADT, but resistance to these inhibitors is inevitable. Resistance mechanisms are thought to occur through increased function of the glucocorticoid receptor (GR), ligand-independent AR splice variants, as well as the activation of other oncogenic pathways that are independent of AR signaling [33,34]. In late stage PC progression, treatment may induce the expansion of phenotypes such as neuroendocrine castration resistant PC (NE-CRPC), which are AR independent and reliant upon specific signaling alterations, including N-Myc expression, Rb loss and p53 mutations [35]. Recent genomic profiling of warm autopsy samples from patients with metastatic castration-resistant prostate cancer (mCRPC) showed that the most frequent genetic alterations occur in PTEN, AR, p53, and the ETS family of transcription factor fusions [36]. Additionally, Taylor et al. identified that the most commonly altered signaling pathways in mCRPC occur in the PI3K, RAS/RAF, and RB signaling axes [37]. Targeting these key driver pathways in PC may allow for the control of resistance to monotherapies and could allow for more effective treatment combinations with immunotherapeutic strategies.

## 3. Changes in Tumor Immunity during Prostate Cancer Progression

During PC progression, there are also increases in tumor immunity, which may occur in a tumor cell autonomous manner or by changes within the patient tumor microenvironment (TME). It is these changes that contribute towards increasing tumor evasion of immune recognition during progression from early to late stage disease, and the supposed reduced response of late stages patients to current immunotherapy approaches [2,38,39]. The stage dependent increase in tumor immunity is supported by the observation that immunotherapies, including Sipuleucel T, work better in earlier stage disease in patients with PC [38,39].

### 3.1. Effects of Inflammation on Disease Initiation and Tumor Immunity

Inflammation in prostate cancer can promote disease initiation, progression and an increase in tumor immune evasion through the recruitment of immunosuppressive cells within the TME [40]. Analysis of patient biopsies from the placebo arm of the Prostate Cancer Prevention Trial (PCPT) showed that chronic inflammation is common in benign tissue surrounding PC and positively associated with high-grade disease [41]. Additionally, an increase in systemic inflammation in PC patient samples has been correlated with a decrease in overall survival (OS) [42], while the use of non-steroidal anti-inflammatory drugs (NSAIDs) correlated with reduced risk of PC development and progression [43]. Taken together, these findings indicate that inflammation promotes PC disease progression. PC inflammation can promote PC through immunosuppression of cells in the TME through several mechanisms: (1) subversion of the T cell-mediated immune response by induction of immunosuppressive myeloid derived suppressor cells (MDSCs) [44], (2) inflammation induced, reversible loss of tumor-specific antigens [45], and (3) pro-inflammatory tumor-associated macrophages (TAMs) [46] that release immunosuppressive cytokines, including vascular endothelial growth factor (VEGF) [47], resulting in increased angiogenesis and metastasis [48,49]. PC preclinical data has shown that induction of VEGF is itself a lymphangiogenic switch, sufficient to promote metastasis in PC cell lines [49]. As well, in preclinical melanoma and colon cancer models, VEGF blockade creates an antitumor immune response by increasing the ratio of effector T cells to immunosuppressive regulatory T cells (Tregs) [50]. In accordance, the combining of VEGF inhibition and immune checkpoint blockade in melanoma leads to superior therapeutic activity compared to either single agent alone [51]. Therefore, targeting inflammation-induced VEGF expression may be a viable strategy to inhibit tumorigenesis and enhance immune checkpoint inhibition in PC patients through a reduction in TME immunosuppression. Indeed, the VEGF inhibitor Bevacizumab has been evaluated in a phase II trail in conjunction with ADT, with results indicating an improvement in relapse-free survival (RFS) compared to ADT alone [52]. Bevacizumab and other inhibitors capable of inhibiting VEGF, such as Sunitinib, will be evaluated for treatment of mCRPC (NCT01803503) [53] and should also be explored in conjunction with T cell-based immunotherapeutic strategies for patients with PC. 

### 3.2. Correlation of an Immunosuppressive Tumor Microenvironment with Prostate Cancer Progression

Corresponding with PC tumorigenesis is a progressive diversification of the TME and an increased abundance of immunosuppressive cells. Oncogenic drivers of tumor epithelia can alter signaling pathways within the TME, thereby leading to immunoresistance. An analysis of PC clinical samples indicates a positive correlation in the infiltration of immunosuppressive cells such as Tregs and myeloid-derived suppressor cells (MDSCs) and disease progression [54]. Interestingly, when compared to cancers that have shown a more robust response to immunotherapies such as melanoma, PCs have a significantly higher number of immunosuppressive cells [55], indicating a potentially greater role in tumor immunity than in other diseases. The acquisition of oncogenic drivers common in PC, as well as treatment-induced alterations in signaling pathways, has the potential to mediate an immunosuppressive TME. For example, in PC patients, an increase in TAMs has been positively correlated with disease stage [56]. ADT can induce infiltration of TAMs into the TME [57,58] through the increased tumor expression of cytokines such as CSF-1 [59] and CCL2 [58]. In accordance, blockade of the CSF-1 receptor in both mouse and human PC cell lines can enhance androgen blockade [59]. 

Changes in MDSCs infiltration is also observed during PC progression [60], suggesting a role for MDSCs in tumor immunosuppression. Genetically engineered mouse models of PC having prostate specific deletion of PTEN [10] alone or with and Smad4 [61] yield aggressive tumors and infiltration of MDSCs. Collectively, these data suggest that loss of PTEN may regulate tumor immunity in a dose-dependent manner and intermediate levels of PTEN within the TME may differentially impact tumor immunity. Other oncogenic events common in late stage mCRPC, such as loss or mutated p53 may also have the potential to enhance the immunosuppressive TME. In preclinical melanoma mouse models, p53 loss of function promotes a MDSC accumulation within the TME [11], while in other mouse models and PC cell lines p53 knockout can increase the production of pro-inflammatory cytokines and promote inflammation through NF-KB activation [62]. Collectively, this data indicates that prostate cancer progression is associated with an increased abundance of immunosuppressive cell types within the TME, and that this increase may be driven in part by the acquisition of oncogenic drivers.

In addition to an increase in immunosuppressive cell types within the TME, alterations in T cell immune checkpoints may also contribute to immunoresistance in PC. Analyses of primary tumor patient samples [63] and enzalutamide-resistant mCRPC [64] indicate that an increase in the expression of the immune checkpoint protein PDL-1 is associated with more aggressive tumors. A similar analysis has demonstrated increases in expression of the immune exhaustion marker TIM-3 with potential correlations with disease stage [65]. Oncogenic alterations common to PC can regulate immune checkpoint protein expression in other disease types. These include the loss of p53 associating with increased PDL-1 expression in NSCLC in vivo mouse models and cell lines [66], Ras-MAPK regulation of PDL-1 expression in triple negative breast cancer (TNBC) [67] and melanoma mouse models and cell lines [68,69], as well as MAPK regulation of TIM-3 expression [70]. These data suggest that the combined use of immune checkpoint inhibitors with clinically viable therapies for targeting oncogenic drivers in advanced PC may be beneficial. 

### 3.3. Targeting Immunosuppressive TME Subtypes in PC

While aggressive PCs can exhibit high levels of expression of immune checkpoint proteins such as PDL-1, variability in expression of these proteins is reported to be considerable [63,71,72]. Therefore, adopting proposed systems of TME classification for use in PC could help to stratify patients into disease stage and TME-specific treatment groups. In their paper, “*Classifying cancers based on T cell infiltration and PD-L1*”, Teng et al. [73] describe four types of TME found in melanoma with different predictive responses to immune checkpoint therapy. These include: Type 1 (TIL^+^; PDL-1^+^), which are predictive of a response to PDL-1/PD-1 immune checkpoint blockade; Type 2 (TIL^−^;PDL-1^−^) and Type 3 (TIL^−^; PDL-1^+^), which are poorly responsive to immune checkpoint blockade due to a lack of TILs; and Type 4 (TIL^+^; PDL-1^−^), in which PDL-1 expression is low and immunosuppression is dominated by other suppressive pathways such as TAMs or MDSCs despite the presence of TILs in the TME [73]. A similar stratification of PC TME subtypes may aid in the selection of appropriate therapeutic strategies. Early stage PCs may include Type 2 and Type 3 TMEs in which immune infiltration and PDL-1 expression are generally lower. Sipuleucel-T and Prostvac can induce immune infiltration into the TME [74,75], and may therefore induce tumor infiltrating lymphocyte (TIL) infiltration in Type 2 PCs and potentially sensitize Type 3 PCs to immune checkpoint blockade. Type 1 PCs would include patients with high PDL-1 and high TIL infiltration associated with advanced disease stage [63,76] and may be responsive to immune checkpoint blockade. Considering this, increases in Type 4 TMEs mediated by a stage-dependent increase in MDSCs, TAMS, and Tregs [60,77,78,79] may account for resistance to immune checkpoint blockade, despite the presence of TILs and expression of PDL-1. Therefore, immune checkpoint monotherapies that only target T cell checkpoints may function poorly despite the presence of TILs and PDL-1, due to a concurrent increase in a Type 4 TME. It will be beneficial to evaluate how oncogenic signaling pathways regulate expansion of immunosuppressive cell types within Type 4 PC to determine if targeting these pathways might sensitize tumors to immune checkpoint blockade through a reduction in Type 4 immunosuppression. Recent works from the laboratory of Ron DePhino have shown that in mouse PC models, chemokine signaling can enhance MDSC recruitment and promote tumor progression [61]. Synergistic treatment results in these models were achieved by combining the dual PI3K/mTOR inhibitor BEZ235 (p110α/γ/δ/β and p70S6K) to target MDSCs in conjunction with CTLA-4 and PD-1 immune checkpoint blockade [80]. Combinatorial strategies such as this that target tumor-mediated chemokine signaling or the use of kinase inhibitors to co-target PC oncogenic drivers, such as VEGF and PI3K, which can also modulate the activity of immunosuppressive cells within the TME, may offer opportunities for immunotherapeutic synergy in a clinical setting.

### 3.4. The Impact of Standard of Care Treatments on Prostate Tumor Immunity

Developing novel strategies to target oncogenic drivers as immunotherapeutic co-targets must take into account the impact of standard-of-care treatments on both disease progression and tumor immunity. 

#### 3.4.1. Effects on Nuclear Receptor Signaling

Increasing evidence suggests that ADT has immunosuppressive influences on PC [57,58,59,81]. Paradoxically, ADT can increase TIL infiltration in PC [81], inducing a short-term increase in T cells within the TME. This increase, however, is paralleled by an expansion of immunosuppressive immune cells such as Tregs [82] and TAMs [59] through an increase in expression of cytokines by tumor cells, including CSF-1 [59]. The recruitment of TAMs by ADT can skew differentiation of naïve CD4^+^ prostate-infiltrating lymphocytes (PILs) towards an immunosuppressive (CD4^+^/FoxP3^+^) Treg phenotype [83] occurring by the stimulation by TAM-secreted cytokines such as IL-6 and TGF-β [84]. Thus, one consequence of ADT is a potential reduced response to immunotherapy as substantiated by the decreased effectiveness of Sipuleucel-T when administered after ADT [85]. Similarly, direct inhibition of AR may also suppress T-cell differentiation and activation [86], and compromise T-cell response [87] occurring through the inhibition of immunostimulatory signaling pathways such as IFN-λ production [87], which in melanoma cell lines can lead to resistance to CTLA-4 blockade [88]. In summary, these findings indicate that the disruption of androgen signaling promotes an immunosuppressive TME with the potential to lessen the effectiveness of certain immunotherapies.

Glucocorticoids (typically low dose prednisone) are used during the course of PC treatment in conjunction with docetaxel or abiraterone acetate but, like androgens, can impact the immune cell content of tumors. Glucocorticoids mediate immunosuppression through interactions with the GR in several ways, including disruption of T cell receptor (TCR) signaling and the induction of immune checkpoint proteins CTLA4, PD-1, and PDL-1 [89,90,91,92,93,94]. In PC, elevated intratumoral synthesis of glucocorticoids can occur through reduced function of the enzyme 11βHSD2 [95], which catalyzes the conversion of the active endogenous glucocorticoid cortisol to its inactive form cortisone [95], as well as the conversion of the active metabolite prednisolone to its inactive form prednisone [96]. Given the immunosuppressive effect glucocorticoids exert on T-cell activation [92], elevated intratumoral levels of glucocorticoids through alterations in steroidogenic pathway enzymes such as 11βHSD2 may lead to a particularly immunosuppressive TME in some PC patients. Interestingly, in the AFFIRM clinical trial for enzalutamide, the use of glucocorticoids in conjunction with enzalutamide yielded worse patient survival outcomes than those receiving enzalutamide alone [34]. This data emphasizes the need for a thorough evaluation of the optimal timing and application of corticosteroids during disease progression, and whether judicious targeting of GR or steroidogenic pathways could present a strategy to delay enzalutamide-resistant tumor progression, as well as sensitize PC patients to immunotherapy through a reduction in glucocorticoid-driven immunosuppression.

#### 3.4.2. The Impact of Chemotherapy and Radiotherapy on Immunotherapy

Chemotherapies (docetaxel, cabazitaxel) are used to treat PC patients who have failed AR targeted therapies. However, these therapies can also have profound effects on the immune system, including acute reductions in NK, T, and B cells [97]. Paradoxically, while treatment with docetaxel may induce lymphocytopenia in patients [97], upon regeneration of immune cell populations, an enhanced anti-tumor immune response can occur through a reduction of immunosuppressive cell types, including MDSC suppression [98] and the selective depletion of Treg cells [99]. Interestingly, in mouse models, the administration of docetaxel after vaccine-based immunotherapy increased the response compared to either treatment alone [100], potentially as a result of increased T cell response and neoantigen presentation [101]. In patient studies, evaluating immune response to taxanes in breast cancer, increased T-Cell and natural killer (NK) activation is also correlated with the use of docetaxel [102]. These results indicate that use of immunotherapy may augment the enhanced immune response seen following the use of standard-of-care chemotherapy. 

Radiotherapy (RT) is used to treat both primary PC (brachytherapy or external beam radiation therapy, EBRT) as well as in late stage mCRPC (Radium-223), and can promote an immunogenic TME through an increase in antigen-presenting cells (APCs) [103]. Radiotherapy can also have an impact on metastases outside of the field of radiation [104] through the abscopal effect also observed in melanoma patients [105]. In preclinical prostate cancer mouse models, EBRT can increase the efficiency of adoptive T cell transfer immunotherapy in a time-dependent manner, with the greatest T cell proliferation and activation occurring at seven weeks following EBRT and tapering off after 14 weeks [106]. This data indicates that the optimal combination of radiotherapy and immunotherapy may occur within a discrete window of time. In clinical trials evaluating the use of radiotherapy in conjunction with immune checkpoint inhibition, mCRPC patients who had progressed after treatment with docetaxel had an increase in clinical activity through combined radiotherapy and immune checkpoint blockade. However, this increase did not translate into a significant extension of OS in patients. [107,108], perhaps due to additional immunosuppressive pressure within the TME. 

Collectively, these observations indicate that standard of care therapies may have both positive and negative consequences on immunotherapy. Appropriate preclinical and clinical trials must be conducted to determine whether combining inhibitors of oncogenic driver pathways with immunotherapeutics should be carried out alone or in conjunction with standard of care treatments. 

## 4. Tumor Autonomous Oncogenic Drivers in Prostate Cancer Progression as Novel Immunotherapeutic Co-Targets during Early, Mid and Late Stage Disease

### 4.1. Early Stage Disease

Standard-of-care therapies for treatment in early disease stage include active surveillance, ADT, surgical intervention, and radiation therapy [109]. Although the vast majority of early stage PCs are manageable with standard-of-care therapies, these treatment options have side effects that may negatively impact quality of life. Early stage PC is driven primarily by AR signaling [110], and immunotherapeutic interventions targeting AR driven disease progression early may provide a more robust immune response prior to the development of an immunosuppressive TME during late stage PC. This is illustrated by the superior activity of Sipuleucel-T [85] and PSA vaccine-based therapies [111] when used in earlier disease stages. Current clinical trials comparing the use of Sipuleucel-T and immune checkpoint inhibitors will reveal if these combinatorial strategies may work either additively or synergistically, and could provide rationale for the incorporation of immune checkpoint inhibition with Sipuleucel-T prior to ADT. Additionally, the use of vaccine-based therapies such as Prostvac may be incorporated in early stage disease to prime the immune response to target PC tumor cells early in progression. Novel strategies that co-target immune system priming for both PSA and prostatic acid phosphatase (PAP) in early stage disease could maximize the immune response to recognize the biomarkers associated with PC progression prior to the enhanced immunosuppressive TME correlated with late stage disease. As the mechanisms underlying immunoresistance in PC are more clearly elucidated, it is tempting to speculate that immunotherapeutic intervention, in early stage, could one day supplant the need for current standard-of-care therapies.

### 4.2. Castration Resistant Prostate Cancer (CRPC)

Failure to control disease progression subsequent to ADT leads to castration-resistant prostate cancer (CRPC). Defining which signaling pathways are sufficient to initiate castration resistance have been best modeled using genetically engineered mouse models of PC. Single allelic loss of the PTEN tumor suppressor through loss of heterozygosity (LOH) may serve as an important transition to invasive disease [32,112] and castration resistance. Complete loss of PTEN leads to activated PI3K/AKT signaling, cell autonomous disease progression, and potential impacts on immunosuppression within the TME. In melanoma cell lines, PTEN loss has been shown to mediate host immune response to cancer through upregulation of immunosuppressive cytokines IL-6, IL-10, and VEGF, as well as through the regulation of PDL-1 expression [9,14]. Conditioned media derived from these PTEN-deficient cell lines also had immunosuppressive effects on dendritic cells subsequently cultured in the media [9]. This dendritic cell suppression could be abrogated by using antibodies to neutralize IL-6, IL-10, and VEGF in the media [9], further suggesting that PTEN loss may enhance immunosuppression. Interestingly, when these cell lines were treated with the PI3K (PI3K-α/β/δ) inhibitor LY294002 and the tyrosine kinase (VEGFR, Raf-1) inhibitor Sorafenib, inhibition of these pathways reduced the expression of immunosuppressive cytokines and the combined effect was greater than either single agent alone [9]. These data indicate that the co-targeting of oncogenic pathways may provide benefits to reduce immunosuppression within the TME. 

In mouse models of melanoma, loss of PTEN impairs T-cell mediated tumor killing through the expression of immunosuppressive cytokines and VEGF, thereby reducing the efficacy of the PD-1 blockade [14]. Accordingly, treatment with PI3K inhibitors in these models was sufficient to enhance sensitivity to both PDL-1 and CTLA-4 checkpoint blockade [14]. Given the consequences of PTEN loss or alterations in the PI3K-AKT signaling axis on immunosuppression in other cancers, targeting PI3K signaling in conjunction with immune checkpoint inhibition as well as VEGF inhibition may offer opportunities for the combination of targeted and immunotherapies in localized CRPC. Indeed, there are a number of inhibitors targeting the PI3K-AKT pathway currently in clinical trials for PC including Ipatasertib (pan-AKT inhibitor, NCT01485861) [113], GSK2636771 (PI3K-β inhibitor, NCT02215096) [114], AZD8186 (PI3K-β/δ, NCT01884285) [115] and AZD5363 [pan-AKT inhibitor, NCT02525068] [116]. Given the frequency of PTEN loss in PC and the effects its loss may have in promoting both tumor progression and TME immunosuppression, targeting the PI3K-AKT pathway in mid stage disease combined with immune checkpoint inhibition may provide enhanced therapeutic benefits. 

Gene fusions of ETS transcription factors are found in over 50% of patients with clinically localized PC [117], and may also serve as a biomarkers for progression from mid to late stage disease [118]. This genomic alteration can promote TME immunomodulation by increasing WNT signaling and NF-kB expression [119,120,121] within the TME. Increased NF-kB signaling is associated with PC progression and can mediate suppression of immunosurveillance by both innate and adaptive immune cells [121,122,123]. In human PC cell lines, ERG-TMPRSS fusions have been shown to drive tumor progression through WNT signaling within the TME [124] corresponding with immune resistance by induction of tumor tolerance in dendritic cells [125]. Given the reported effects of WNT signaling on tumor progression and dendritic cell tumor tolerance, inhibiting the WNT pathway, particularly in conjunction with dendritic cell-based therapies such as Sipuleucel-T, could offer enhanced therapeutic benefit in patients with ETS family gene fusions. Clinical trials using the WNT pathway inhibitor LGK974 [NCT01351103] alone and in conjunction with anti PD-1 immune checkpoint inhibition in patients with melanoma, B-Raf serine/threonine kinase (BRAF) mutant colorectal cancer and triple negative breast cancer are currently recruiting [126]. The use of these agents, combined with standard-of-care therapies and the targeted inhibition of the PI3K-AKT pathway in patients with PTEN loss and ERG-TMPRSS fusions, may be a viable strategy to delay tumor progression and promote immunotherapeutic sensitivity in patients prior to progression to late stage PC. Collectively, these data indicate that an enhanced therapeutic response may be achieved through combined immunotherapy and inhibition of common oncogenic drivers found at the onset CRPC, which may contribute to cell autonomous cancer progression and tumor immune evasion in CRPC.

### 4.3. Metastasis

When disease has progressed to metastases after standard-of-care therapies have failed, mCRPC is lethal. In this late stage disease, alterations in DNA damage repair mechanisms [127] can contribute to increasing genomic instability and mutational divergence in patients. Standard-of-care therapies can also induce the expansion of enzalutamide-resistant subclones that are mutationally divergent from clonal populations in earlier disease stage [35,128]. Despite the increasing complexity and heterogeneity of tumors in late stage disease, targeting common mutations that have immunomodulatory potential may contribute to a reduction of immunosuppression within the mCRPC TME, and increase sensitivity of tumors to immune checkpoint blockade. Two actionable mutations common in mCRPC that may impact tumor immunity are alterations to p53 and speckle-type POZ protein (SPOP) mutations. 

Loss of function mutations in p53 are found in more than 50% of mCRPC patients and are mutationally enriched in mCRPC compared to primary tumor samples [36]. Loss of function of p53 can impact immunosuppression through increased PDL-1 expression and activation of NF-kB signaling [129,130], an increase in immunosuppressive MSDCs [11] and an induction of inflammatory cytokines [62]. Targeted therapies designed to reactivate mutant p53 pathways such as APR-246 are currently in clinical trials (NCT00900614, NCT02098343) and have shown the potential for synergism with chemotherapy in ovarian cancer [131,132]. Given the capacity for p53 to regulate immune checkpoint signaling, utilizing strategies to reactivate p53 may offer combinatorial strategies to sensitize tumors to immune checkpoint blockade through the modulation of PDL-1. Mutations in the SPOP DNA damage repair protein have also been identified as a genomic alteration occurring in as many as 15% of patients with mCRPC [133]. SPOP mutations promote PC progression through an increase in genomic instability, but may also sensitize patients to poly ADP ribose polymerase (PARP) inhibitors, which target alternate mechanisms of DNA damage repair [134]. Evidence from research in ovarian cancer suggests that PARP inhibition can synergize with CTLA-4 checkpoint blockade [135], and may therefore be an attractive combinatorial therapy with immune checkpoint therapies in PC. Numerous PARP inhibitors are in clinical trials for PC, including olaparib and rucaparib [136,137], which should be tested in conjunction with immunotherapeutic strategies. Utilizing SPOP mutations as biomarkers, the co-targeting of DNA damage repair mechanisms with PARP inhibitors in conjunction with immune checkpoint blockade [135,138] could offer an additional viable co-targeting strategy for patients with this disease profile. 

In addition to combining targeted therapies with immune checkpoint inhibition in late stage disease, other immunotherapeutic approaches can target these mutations to enhance immune cell tumor recognition. Recently, T cell adoptive transfer therapies targeting mutant K-Ras proto-oncogene/GTPase (KRAS) have shown remarkable efficacy in the treatment of human colorectal cancer [139]. Utilizing such therapies to target neoepitopes generated by mutations to p53 and SPOP mutations in mCRPC could provide additional treatment options for late stage disease with the potential to reduce immunosuppression within the PC TME. 

The RAS/MAPK pathway is activated in many PC metastasis, is associated with reduced PC survival [140] and can cooperate with PTEN loss to promote metastasis in PC mouse models [141]. In addition to promoting tumor progression, MAPK signaling can also promote tumor immunoresistance. Preclinical mouse models of melanoma have shown that the targeted inhibition of MAPK signaling can lead to an improved immune response through enhanced CD4 and CD8 activation, attenuation of Treg-mediated immunosuppression, and superior dendritic cell antigen presentation [13]. In mouse models of multiple myeloma, activated MAPK signaling has been shown to suppress dendritic cells [142]. However, in these models, inhibition of MAPK signaling restored dendritic cell activity and induced cytotoxic T lymphocyte responses to tumor cells [142]. Additional studies in preclinical mouse models of colon cancer and triple negative breast cancer also support the rationale for targeting tumor immune evasion through the inhibition of MAPK signaling. In these studies, MAPK/ERK kinase (MEK) inhibition-enhanced T cell activity and promoted an anti-tumor immune response in conjunction with PDL-1 inhibition [67,143]. Collectively, these studies indicate that the inhibition of RAS/MAPK signaling may provide a viable strategy to target both disease progression and immunosuppression in conjunction with immunotherapy in PC. Clinical trials targeting MAPK pathway kinases such as trametinib (MEK1/2, NCT02881242) and eFT508 (MNK1/2, NCT02605083) are currently recruiting for patients with PC [144,145], and future evaluation of whether these agents may sensitize mCRPC patients to immunotherapies may be warranted.

**Figure 1:**
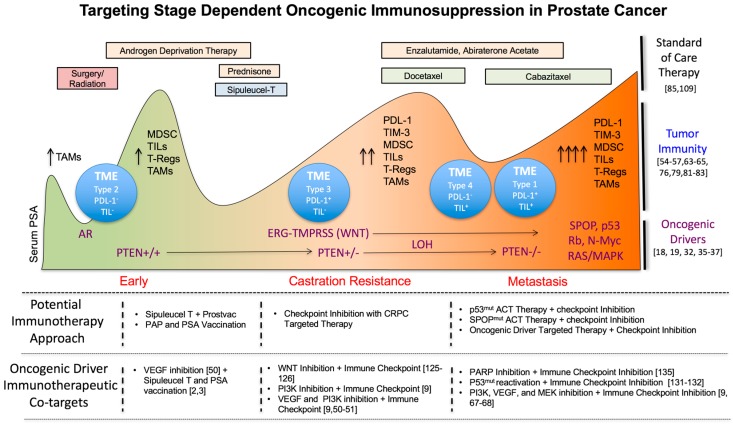
Enhancing prostate cancer therapy by combining immunotherapy with targeted inhibition of oncogenic driver pathways. Oncogenic drivers of progression promote tumor heterogeneity and tumor immune resistance. Targeting driver pathways may enhance the effectiveness of immunotherapies when delivered with respect to the stage of progression and patient tumor microenvironment (TME) subtypes. ACT = adoptive cell transfer.

Figure 1 below summarizes potential targeted therapy and immunotherapeutic combinations with respect to standard of care therapies, patient serum PSA, and tumor immune microenvironment during PC progression. Co-targeting oncogenic driver pathways that may contribute to a stage dependent increase in tumor immunoresistance during PC progression, could offer opportunities for enhanced therapeutic benefit when used in conjunction with immunotherapies in patients with PC.

## 5. Discussion

The heterogeneity of PC and increasing occurrence of treatment-induced lineages demands the development of progression and patient-specific immunotherapies. In other genetically heterogeneous cancer types such as multiple myeloma, a treatment approach using a combination of agents to target multiple pathways of disease progression has shown dramatic efficacy in disease management [146,147]. The identification of multi-agent treatment combinations for disease subtypes may prove to be equally effective in the treatment of PC. While multiple agents are available for clinical evaluation, the potential toxicity associated with the use of combinatorial agents must be assessed. Large-scale clinical trials are not capable of quickly and economically comparing the multitude of different potential treatment combinations for PC. Thus, it will be increasingly important that robust preclinical in vivo models be developed for PC as tools to quickly and comprehensively asses the effects of agent combinations targeting multiple drivers of tumor progression and immunoediting mechanisms for expeditious translation into a clinical setting. 

Given the complexity of how multiple driver pathways may impact tumor progression and immune response, these model systems must (1) accurately represent combinations of oncogenic drivers as seen in genomic and gene expression analysis in patient populations; (2) comprehensively evaluate how these oncogenic combinations impact both tumor progression and immunosuppression in the TME; and (3) be quickly adaptable to generate oncogenic combinations in an immune-competent in vivo model system. 

The use of CRISPR-Cas9 technologies, capable of efficiently generating targeted loss and gain of function mutations, has been recently described in Cas9 knock-in mouse models of lung cancer [148]. Utilizing The Cancer Genome Atlas data sets for lung cancer, common mutational drivers were identified in patients and recreated in a tissue-specific manner in the knock-in mice. Model systems such as this could provide a way to rapidly and specifically generate combinations of oncogenic drivers implicated in PC disease progression. Analysis of these PC-specific model systems utilizing high throughout applications such as mass cytometry (CyTOF) will allow for a more complete characterization of the ways in which the acquisition of oncogenic drivers during PC disease progression can modulate immunosuppressive cell populations within the PC TME, and will provide a platform for in vivo targeting of multiple oncogenic and immune checkpoint axes to assess translational relevance. 

Using these strategies, it may be possible to expand the number of patients who respond to immunotherapy in a disease that remains the second leading cause of cancer-related death among males in the United States [149].

## Abbreviations

ACTs	adoptive cell transfer therapies
APCs	antigen presenting cells
AR	androgen receptor
CRISPR	clustered regularly interspaced short palindromic repeats
CRPC	castration resistant prostate cancer
EBRT	external bean radiation therapy
EMT	epithelial-mesenchymal transition
ERG	ETS-related gene; GR glucocorticoid receptor
mCRPC	metastatic castration resistant prostate cancer
MDSC	myeloid derived suppressor cell
NE-CRPC	neuroendocrine castration resistant prostate cancer
NK	natural killer cell
NSAIDs	non-steroidal anti- inflammatory drugs;
OS	overall survival
PAP	prostatic acid phosphatase
PCPT	prostate cancer prevention trial
PILS	prostate infiltrating lymphocytes
PSA	prostate specific antigen
RT	radiotherapy
SPOP	speckle type poz protein
TAM	tumor-associated macrophage
TILs	tumor infiltrating lymphocytes
TME	tumor microenvironment
TNBC	triple negative breast cancer
